# Evaluation of lymph node numbers for adequate staging of Stage II and III colon cancer

**DOI:** 10.1186/1756-8722-4-25

**Published:** 2011-05-28

**Authors:** Chandrakumar Shanmugam, Robert B Hines, Nirag C Jhala, Venkat R Katkoori, Bin Zhang, James A Posey, Harvey L Bumpers, William E Grizzle, Isam E Eltoum, Gene P Siegal, Upender Manne

**Affiliations:** 1 Departments of Pathology, University of Alabama at Birmingham, Birmingham, AL 35294, USA; 2 Jiann-Ping Hsu College of Public Health, Georgia Southern University, Statesboro, GA 30460, USA; 3 Department of Pathology and Laboratory Medicine, University of Pennsylvania, Philadelphia, PA 19104, USA; 4 Department of Biostatistics, University of Alabama at Birmingham, Birmingham, AL35294, USA; 5 Department of Medicine, University of Alabama at Birmingham, Birmingham, AL35294, USA; 6 Department of Surgery, Morehouse School of Medicine, Atlanta, GA 30310, USA; 7 Comprehensive Cancer Center, University of Alabama at Birmingham, Birmingham, AL 35294, USA

**Keywords:** Colon cancer, Clinical outcomes, Lymph nodes, Stage II, Stage III

## Abstract

**Background:**

Although evaluation of at least 12 lymph nodes (LNs) is recommended as the minimum number of nodes required for accurate staging of colon cancer patients, there is disagreement on what constitutes an adequate identification of such LNs.

**Methods:**

To evaluate the minimum number of LNs for adequate staging of Stage II and III colon cancer, 490 patients were categorized into groups based on 1-6, 7-11, 12-19, and ≥ 20 LNs collected.

**Results:**

For patients with Stage II or III disease, examination of 12 LNs was not significantly associated with recurrence or mortality. For Stage II (HR = 0.33; 95% CI, 0.12-0.91), but not for Stage III patients (HR = 1.59; 95% CI, 0.54-4.64), examination of ≥20 LNs was associated with a reduced risk of recurrence within 2 years. However, examination of ≥20 LNs had a 55% (Stage II, HR = 0.45; 95% CI, 0.23-0.87) and a 31% (Stage III, HR = 0.69; 95% CI, 0.38-1.26) decreased risk of mortality, respectively. For each six additional LNs examined from Stage III patients, there was a 19% increased probability of finding a positive LN (parameter estimate = 0.18510, p < 0.0001). For Stage II and III colon cancers, there was improved survival and a decreased risk of recurrence with an increased number of LNs examined, regardless of the cutoff-points. Examination of ≥7 or ≥12 LNs had similar outcomes, but there were significant outcome benefits at the ≥20 cutoff-point only for Stage II patients. For Stage III patients, examination of 6 additional LNs detected one additional positive LN.

**Conclusions:**

Thus, the 12 LN cut-off point cannot be supported as requisite in determining adequate staging of colon cancer based on current data. However, a minimum of 6 LNs should be examined for adequate staging of Stage II and III colon cancer patients.

## Background

In 2010, an estimated 51,370 deaths from colorectal cancer (CRC) are expected to have occurred, accounting for 9% of all cancer deaths in the USA [1]. For CRC patients, the stage of the disease predicts long-term survival and is weighed in designing treatments [2]. The acquisition of a single positive lymph node (LN) identifies Stage III patients, and the prognosis worsens as the number of involved LNs increases [3]. These patients are characterized by a high recurrence rate but may be benefitted by adjuvant chemotherapy [4-6]. Currently, due to conflicting results from clinical trials and population-based studies, the role of adjuvant chemotherapy for Stage II patients remains controversial [6]. Some investigators, however, recommend chemotherapy for all high-risk Stage II CRC patients, including those with inferior LN recoveries and with peritoneal involvement, extramural vascular invasion, tumor perforation and/or tumor obstruction [3,7].

LN involvement is the key factor that determines the stage and prognosis for CRCs [8]. Nevertheless, LN positivity alone does not identify all patients with a poor prognosis, as 20 to 40% of patients with Stage II (LN-negative) disease die of their cancers [9,10]. In population-based studies, the percentages of CRCs in Stages II and III are approximately 40% and 30%, with 5-year, cancer-specific survival rates ranging between 50-80% and 30-60%, respectively [11,12]. The proportion of Stage III tumors may, however, be higher than reported because of missing LN metastases due to inadequate examination and resulting under-staging [13]. The suggested minimum number of LNs to be examined to stage these patients has ranged between 6 and 20 [11,14-18]. The World Congress of Gastroenterology proposed examination of a minimum of 12 LNs for classification of tumors as Stage II [19]. In the USA, the American Joint Committee on Cancer (AJCC), the American Society of Clinical Oncology (ASCO) and the College of American Pathologists (CAP), American College of Surgeons (ACoS), Commission on Cancer (CoC), and the National Comprehensive Cancer Network (NCCN) have also recommended examination of at least 12 LNs to assign Stage II disease [8,20,21].

Several institutional and population-based studies showed a survival benefit associated with increasing numbers of LNs examined from Stage II and Stage III CRC patients [22-25]. The origin of the database (Surveillance Epidemiology and End Results, SEER, versus NCCN) also influenced the findings of LN examination on patient prognosis [17]. In one investigation, increased numbers of LNs examined was associated with improvements in overall survival and relapse-free survival for Stage II but not in Stage III patients [26]. Goldstein [27] reported that the predictive probability of finding positive LNs increased with increasing numbers of LNs examined. In contrast, Bui et al [22] and Wong et al [15] found no substantial increase in LN positivity with increased numbers of examined LNs.

The recommendation by AJCC, ASCO, ACoS-CoC, CAP, and NCCN, that examination of ≥12 LNs is sufficient to stage a patient with CRC would seem to end the debate yet anecdotal evidence suggests these recommendations may not be followed. To determine how many LNs should be examined from Stage II and III patients with colon cancer, we evaluated a consecutive retrospective cohort and assessed cancer-specific mortality and recurrence. We also attempted to derive a minimum number of LNs needed to stage patients appropriately and thus to minimize under-staging.

## Methods

### Patients

This investigation was approved by the Institutional Review Board and Bioethics Committee of the University of Alabama at Birmingham (UAB). This cross-sectional study was comprised of Stage II and III cancer patients who underwent surgery for adenocarcinoma of the colon at UAB Hospital from 1981-2002. Follow-up ended in December, 2010. The initial study population consisted of 566 patients. To minimize the influence of familial/hereditary CRCs, patients < 45 years old (n = 23) were excluded, as were those with missing LN information (n = 31). Patients who died within one week of surgery (n = 12) and those who received neoadjuvant chemotherapy (n = 8) were also excluded. Two were removed due to missing tumor grade information. The final study population was 490. In this study, only about 22% (50 of 230) of patients with Stage III disease had received adjuvant chemotherapy for various clinical reasons, and the treatment information was accounted for in the survival analyses.

### Study design

Three pathologists (CS, NCJ, and WEG) extracted the pathologic features from pathology reports and confirmed by reviewing hematoxylin and eosin stained sections. CRCs were classified by the tumor-node-metastasis (TNM) method and staged according to the AJCC system [21]. Tumor grade was recorded as well differentiated, moderately differentiated, poorly differentiated, or unknown; no tumors were graded as undifferentiated. Well and moderately differentiated tumors were designated as "low" grade, and poorly differentiated tumors as "high" grade [8]. Tumor size was also obtained, and a dichotomous variable was created (≥ 5 and < 5 cm).

Demographic, clinical, and patient information regarding age at the time of surgery, gender, race, surgery date, and adjuvant chemotherapy was obtained from medical records. Age was categorized as < 65 and ≥ 65 years. Subjects were classified as non-Hispanic African American, or non-Hispanic Caucasian American, based on self-identification. Patients who had adjuvant treatment were categorized as "yes" if they received any 5-fluorouracil-based chemotherapy.

### Statistical analysis

A nominal categorical variable, including the current recommendation of examining 12 LNs, was created for the number of LNs examined based on a quartile distribution. Patients were categorized by the number of LNs examined at surgery into four groups: 1-6, 7-11, 12-19, and ≥ 20. Survival time was calculated from the date of surgery until either death, the termination date of the study, or the last date of contact for patients who were still alive at the end of the study. The primary events of interest were colon cancer-specific death and recurrence of disease. All reported *P *values were two-sided; statistical significance was defined as *P *< 0.05. All analyses were performed with SAS statistical software, version 9.2.

The chi-square (χ^2^) statistics for categorical variables and the t-test for continuous variables were used to assess differences with respect to vital status, demographics along with tumor-related and clinical variables according to tumor stage. Log-rank tests and Kaplan Meier survival curves [28] were used to compare Stage III pN1 patients with Stage III pN2 patients for colon cancer-specific or disease-specific survival (DSS). The type I error rate for each test was controlled at <0.05. For Stage II and III patients, hazard ratios (HRs) for the bivariate association between the numbers of LNs obtained and other covariates with death due to colon cancers were assessed separately. From the bivariate analysis, all variables that were associated with cancer-specific mortality and risk of recurrence at *P *< 0.20 were entered into the initial multivariable model containing the number of LNs collected as a categorical variable. To obtain the final model for cancer-related mortality, the least significant variable was removed in a step-wise manner. The association between LN examination and recurrence (at 2 and 5 years) or cancer-specific survival was obtained with the overall survival as well as 5-year cancer-specific survival and risk of recurrence. The final multivariable models for survival and recurrence were used separately to obtain HRs for the association between the numbers of LNs examined and cancer-specific survival and risk of recurrence. These multivariable, stage-specific models were adjusted for age, race, gender, treatment, and tumor location, size, and grade to assess the cancer-specific survival or risk of recurrence. For LN-positive patients, the association between the number of LNs examined (continuous) and the number of positive LNs found was assessed. For stage III (LN-positive) patients, linear regression was used to estimate the association between the number of LNs examined (continuous) as a predictor for the number of positive LNs. The linear regression equation to obtain parameter estimation was: *Y = β_0 _+ β_1_X_1 + _β_2_X_2 _+ E(Y *= number of positive lymph nodes, *β_1 _*= the number of lymph nodes examined, and *β_2 _*= covariate, *X_1 _= *the value of number of lymph nodes examined, *X_2 _= *is the value of a covariate, and E = error term). For Stage III patients, the probability of a tumor being classified as pN2 (≥ 4 positive LNs) was obtained for the four categories of LNs.

## Results

### The characteristics of the study population and their cancers

The median age of the study population was 68 (45 to 99 years). As shown in Table 1, Stage III patients were younger (< 65: n = 98, 42.6%; *P *= 0.02) than Stage II patients (n = 85, 32.7%). There were more patients with larger tumors in Stage II (≥ 5 cm: n = 144, 55.4%; *P *= 0.04) than in Stage III (n = 106, 46.1%). In accordance with current treatment recommendations, more Stage III patients received adjuvant chemotherapy (n = 50, 21.7%; *P *< 0.0001).

**Table 1:** Characteristics of the study population (N = 490)

	*StageII (n = 260, 53.1%)*		*Stage III (n = 230, 46.9%)*		
*Variable*	*n*	*(%)*	*n*	*(%)*	*P value*
Age (years)					0.024
< 65	85	32.7	98	42.6	
≥ 65	175	67.3	132	57.4	
					
Sex					0.959
Male	134	51.5	118	51.3	
Female	126	48.5	112	48.7	
					
Race					0.202
Caucasian Americans	165	63.5	133	57.8	
African Americans	95	36.5	97	42.2	
					
Tumor grade					0.380
Low	216	83.1	184	80.0	
High	44	16.9	46	20.0	
					
Tumor location					0.521
Distal	100	38.5	95	41.3	
Proximal	160	61.5	135	58.7	
					
Tumor size (cm)					0.040
< 5	116	44.6	124	53.9	
≥ 5	144	55.4	106	46.1	
					
Adjuvant chemotherapy					< 0.0001
No	237	91.2	180	78.3	
Yes	23	8.8	50	21.7	
					
Status					< 0.0001
Alive	89	34.2	59	25.6	
Death due to colon cancer	77	29.6	123	53.5	
Death due to other causes	94	36.2	48	20.9	
					
Recurrence (years)					0.048
No	209	80.4	163	70.9	
≤ 2	35	13.5	47	20.4	
> 2	16	6.1	20	8.7	
					
Number of LNs harvested					0.615
1-6	55	21.2	38	16.5	
7-11	63	24.2	57	24.8	
12-19	75	28.8	73	31.7	
≥ 20	67	25.8	62	27.0	

There was a significant difference between Stage II and Stage III according to the vital status (*P *< 0.0001). As compared to Stage II patients (n = 77, 29.6%), more Stage III patients died due to colon cancer (n = 123, 53.5%). There were more recurrences within two years among Stage III patients compared to Stage II patients (*P *= 0.048). However, there was no stage difference according to the number of LNs extracted relative to gender, race, or tumor grade.

### The association between the number of LNs collected and colon cancer recurrence

Compared to patients with <12 LNs identified, collection of ≥12 LNs was not significantly associated with recurrence at 2 or 5 years, as determined by multivariate analyses of Stage II and III colon cancers (Table 2). For Stage II patients, the higher categories of LNs obtained were associated with a decreased risk of recurrence, although only the ≥ 20 category approached significance, with a 67% decreased risk of recurrence within 2 years (HR = 0.33; 95% CI, 0.12-0.91). The stage-wise association between LNs harvested and 5-year recurrence, however, was not statistically significant (Table 2). For Stage III patients, there was no relationship between increasing numbers of LNs examined with cancer recurrence (Table 2).

**Table 2:** Multivariate analyses of numbers of LNs obtained and recurrence of colon cancer at 2 and 5 years

	***Adjusted***^***a ***^***HRs (95% C.I.)***	
*LNs extracted*	*Stage II*	*Stage III*
		
Recurrence in 2 years		
		
Current guideline		
< 12	ref	ref
≥ 12	0.62 (0.32, 1.22)	1.27 (0.67, 2.40)
		
Quartiles		
1-6	ref	ref
7-11	0.72 (0.29, 1.79)	1.49 (0.52, 4.26)
12-19	0.63 (0.26, 1.52)	1.54 (0.55, 4.34)
≥ 20	0.33 (0.12, 0.91)	1.59 (0.54, 4.64)
		
Recurrence in 5 years		
		
Current guideline		
< 12	ref	ref
≥ 12	0.67 (0.37, 1.24)	1.16 (0.67, 1.99)
		
Quartiles		
1-6	ref	ref
7-11	0.64 (0.27, 1.51)	1.24 (0.53, 2.91)
12-19	0.62 (0.26, 1.52)	1.23 (0.53, 2.84)
≥ 20	0.47 (0.20, 1.11)	1.44 (0.61, 3.42)
		

HR, hazard ratio; CI, confidence interval.

^a^Adjusted for age, race, and tumor grade. The Stage III model was also adjusted for chemotherapy status and the number of positive LNs.

**Figure 1:**
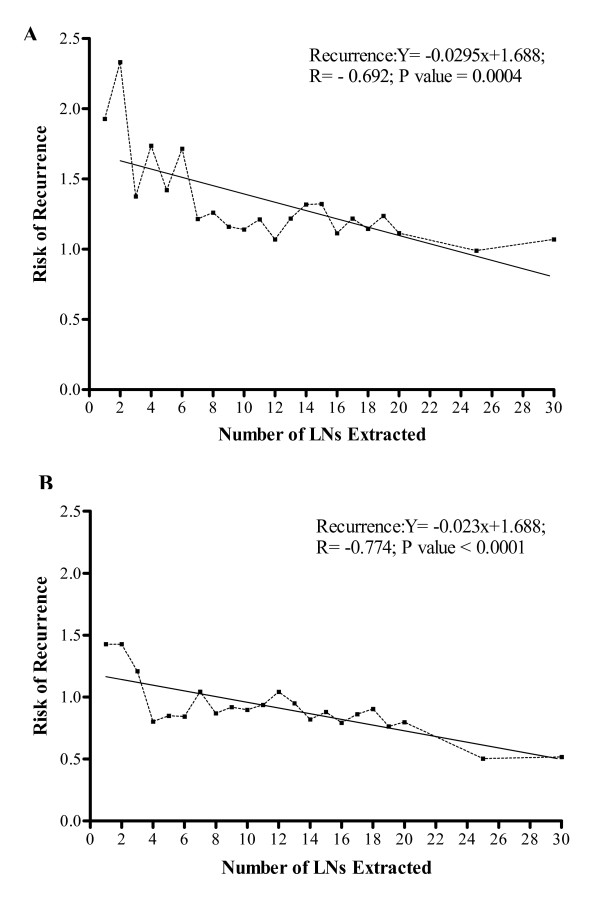
**Overall risk of recurrence in Stage II (A) and III (B) colon cancers**. Linear regression analysis of data from Stage II & III colon cancer patients demonstrates a decreasing risk of recurrence with increasing numbers of LNs identified. Note that Stage II & III patients with < 6 LNs harvested had the highest risk of recurrence; however, collection of 6 to 19 LNs resulted in a similar risk of recurrence. Collection of >20 LNs conferred a significantly reduced risk of recurrence.

The rates of recurrence decreased with increases in the number of LNs removed for both Stage II (*R *= -0.692, p = 0.0004) (Figure 1A) and III (*R *= -0.774, p < 0.0001) (Figure 1B) patients; however, for Stage II and III colon cancer patients, there was no statistically significant difference in the rates of recurrence after the collection of 6 - 19 LNs (Table 2).

### The association between the number of LNs obtained and disease-specific survival

As noted for recurrence at 2 years, multivariate analyses showed that collection of 12 LNs as the cutoff was not significantly associated with disease-specific survival (DSS) for Stage II (HR = 0.61; 95% CI, 0.37 - 1.00) or Stage III (HR = 0.97; 95% CI, 0.64 - 1.46) (Table 3) patients. Multivariate analyses according to the categorical variables, showed, however, that, compared to the category of 1-6 LN retrieved, the three higher categories (7-11, 12-19, ≥ 20) exhibited an improved 5-year and overall DSS. The ≥ 20 category had significantly better survival than those with <6 LNs in Stage II (5 years-HR = 0.42; 95% CI, 0.20 - 0.90; overall- HR = 0.45; 95% CI, 0.23 - 0.87) but not for Stage III (5 years-HR = 0.74; 95% CI, 0.39 - 1.40; overall-HR = 0.69; 95% CI, 0.38 - 1.26) (Table 3).

**Table 3:** Bivariate and multivariate associations of number of lymph nodes harvested with 5-year and overall colon cancer-specific survival

	*Un-adjusted HRs (95% C.I.)*		***Adjusted***^***a ***^***HRs (95% C.I.)***	
*LNs extracted*	*Stage II*	*Stage III*	*Stage II*	*Stage III*
				
5 Years DSS				
				
Current guideline				
< 12	ref	ref	ref	ref
≥ 12	0.65 (0.40, 1.07)	1.13 (0.77, 1.64)	0.61 (0.37, 1.00)	0.97 (0.64, 1.46)
				
Quartiles				
1-6	ref	ref	ref	ref
7-11	0.93 (0.60, 1.43)	1.64 (0.91, 2.94)	0.85 (0.43, 1.67)	0.87 (0.41, 1.85)
12-19	0.89 (0.58, 1.34)	0.97 (0.55, 1.71)	0.68 (0.35, 1.32)	0.97 (0.55, 1.70)
≥ 20	0.54 (0.35, 0.83)	1.02 (0.57, 1.82)	0.42 (0.20, 0.90)	0.74 (0.39, 1.40)
				
Overall DSS				
				
Current guideline				
< 12	ref	ref	ref	ref
≥ 12	0.69 (0.44, 1.07)	1.09 (0.76, 1.56)	0.65 (0.42, 1.02)	0.95 (0.64, 1.40)
				
Quartiles				
1-6	ref	ref	ref	ref
7-11	0.69 (0.37, 1.28)	0.84 (0.48, 1.48)	0.75 (0.40, 1.40)	0.83 (0.47, 1.47)
12-19	0.68 (0.38, 1.24)	1.01 (0.60, 1.70)	0.67 (0.37, 1.22)	0.94 (0.56, 1.59)
≥ 20	0.45 (0.23, 0.87)	0.94 (0.55, 1.61)	0.45 (0.23, 0.87)	0.69 (0.38, 1.26)
				

DSS, disease-specific survival; HR, hazard ratio; CI, confidence interval.

^a^Adjusted for age, race, and tumor grade. The Stage III model was also adjusted for chemotherapy status and the number of positive LNs.

### The association between the number of LNs retrieved with LN positivity in Stage III colon cancer

The number of positive LNs examined was obtained for Stage III patients. As determined by linear regression analysis, each additional LN collected resulted in a 19% increased probability of collecting a positive LN (parameter estimate = 0.1851, p < 0.0001). Therefore, collection of six additional LNs resulted in identification of one additional positive LN (1/0.1851 = 5.4).

### The association between the number of LNs obtained with the probability of identifying pN_2 _tumors in stage III colon cancer

**Figure 2:**
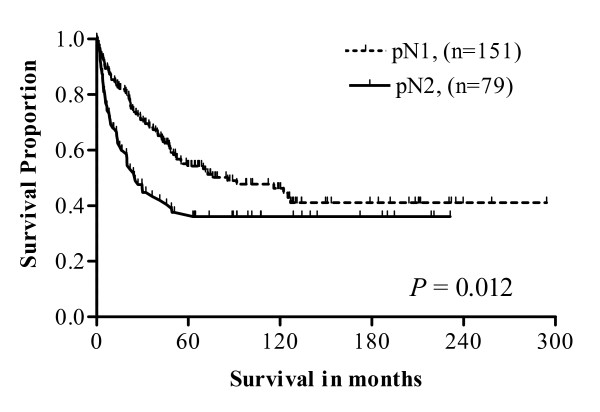
**Survival in pN1 *vs*. pN2 Stage III colon cancers**. Kaplan-Meier survival curves demonstrating significant difference in disease-specific survival between Stage III patient groups of pN1 and pN2.

Logistic regression was utilized to obtain the predictive probability (PP) of obtaining ≥ 4 positive LNs (pN_2 _designation) according to the number of LNs obtained, after adjustment for other confounders. Patients with 1-6 LNs collected had an 18% (PP = 0.184) chance of having a pN_2 _tumor. Patients with 7-11 and 12-19 nodes obtained had probabilities of 37% (PP = 0.370) and 38% (PP = 0.382), respectively. Patients with ≥ 20 LNs extracted had a 43% chance (PP = 0.433) of having a pN_2 _tumor (data not shown). Analysis of Stage III CRCs based on the status of nodal involvement (pN_1 _versus pN_2_) demonstrated no significant difference in the rate of recurrence within 2 (HR = 2.43, 95% CI, 1.37 - 4.32) or 5 years (HR = 2.06, 95% CI, 1.26 - 3.39) (data not shown); however, patients with pN_2 _colon cancers had a lower survival than pN_1 _patients (log-rank p = 0.012) (Figure 2).

## Discussion

For both Stage II and III colon cancer patients, increased numbers of LNs retrieved were associated with reduced risk of recurrence and improved cancer-specific survival. Identification of ≥20 LNs correlated significantly with reduced risk of recurrence and mortality of Stage II but not Stage III patients. For Stage III patients, collection of six additional LNs resulted in identification of one additional positive LN, and the probability of finding patients with pN_2 _nodal stage increased with increasing numbers of LNs examined.

LN involvement determines the pathologic stage and forms the basis for selection of patients for adjuvant therapy [8]. Although Stage II disease, which has a relatively good prognosis, is characterized by the absence of LN involvement, about one third of these patients experience recurrences, due either to missed micro-metastases or to aberrant drainage of LNs beyond the field of resection, leading to under-staging [3,9,12]. Furthermore, for Stage III patients, inadequate LN recognition is associated with a poorer prognosis [15,22], and increased LN collection has a favorable effect on prognosis [29-31]. Thus, diligent searches for LNs are needed for accurate assessment of nodal status and for correct assignment of stage.

The AJCC, ASCO, CAP, ACoS-CoC, and NCCN have recommended that a minimum of 12 LNs be examined in order to rule out metastases via the lymphatic system to nodal tissues [8,20,21]. This recommendation, however, is not widely practiced. Only 58% of those in the SEER database had ≥12 LNs harvested [17], and the NCCN database review documented a 60% failure rate in achieving resection of 12 LNs among various USA hospitals [18]. Various minimum numbers of LNs harvested (range: 6 to 40) from colon resections, have been suggested for adequate staging of colon cancer patients [15,22,26,32].

An increased LN examination confers a survival benefit, especially for stage II disease [14,27,32-34]. In the current investigation, however, examination of 12 LNs showed no significant survival benefit. By disease stage, there was a 55% and 31% reduced risk of cancer-specific mortality for Stage II and III patients, respectively, for those with ≥ 20 LNs examined. The 5-year survival of Stage II cases was 54.9%, whereas the survival of those who had ≥ 9 LNs examined after surgery was 79.9% [35]. The 5-year survival of Stage II patients who had ≤ 8 LNs examined was similar to that for Stage III patients (51.8%). Sixteen of 17 studies of Stage II and 4 of 6 studies of Stage III showed improved patient survival with increased number of LNs examined [16]. In contrast, for Stage III patients, the number of LNs examined did not serve as a prognosticator [36]. The demonstration of increased mortality associated with the examination of ≤ 6 LNs, compared to > 6, especially in Stage II patients, is in concordance with other reports [16,24,27].

With tumor recurrence as the outcome for Stage II patients, all higher categories of LN collections showed a decreased risk, but only the ≥ 20 category approached significance, with a 67% decreased risk of recurrence within 2 years after surgery. In contrast, for Stage III patients, there was no relationship between increasing numbers of LNs examined with colon cancer recurrence. A low risk of recurrence for patients with ≥ 14 LNs examined compared to smaller numbers was reported earlier [32]. There was a significant difference in recurrence with the number of LNs examined. Further, for pN_1 _and pN_2 _patients, disease-free survival improved as more LNs were removed, but there was no such association for node-negative patients. The impact of LN ratio (ratio of tumor-infiltrated nodes to total number of harvested LNs) on 3-year, disease-free survival was more prominent for patients with > 12 LNs examined [37].

In general, examination of an increased number of LNs results in greater chances of identifying LN metastases, thus minimizing under-staging [14,27,32-34]. In our investigation of patients with Stage III disease, for each additional LN collected, there was 19% increased probability of finding a positive LN. Thus, collection of six additional LNs resulted in finding one additional positive LN. In a mathematical model, the predictive probability of identifying single LN metastases was 0.25 if 12 LNs were examined and 0.46 if 18 LNs were examined [27]. Since the probability of LN positivity increases as the number examined increases, there is no minimum number that reliably stages all patients [27,33]. Higher LN counts, however, do not always correlate with increased rates of nodal positivity [22].

The accuracy of staging depends on multiple factors, including those that are modifiable (e.g., surgeon and pathologist) and un-modifiable (e.g., age, obesity, and socioeconomic status of the patient and anatomic location of the tumor) [16,38,39]. Pathologists encounter challenges in adequate LN retrieval. In Stage II colon cancer, the age of the patient, tumor size, specimen length, use of a structured pathology template, and academic status of the hospital are predictors of LN collection [40]. Up to 70% of metastases are found in LNs that are < 5 mm in diameter and hence likely to be missed on routine visualization or palpation [41]. Another challenge for pathologists relates to micro-metastases or isolated tumor cells that are missed in routine histological examinations. Although immunohistochemistry and polymerase chain reactions to identify cytokeratin and carcinoembryonic antigen [42] have been used to highlight malignant cells, the prognostic significance of LNs containing such micro-metastases is uncertain [35]. Targeted LN examination by mapping of the most proximal LN (sentinel LN) improves the staging accuracy for colon cancer [43,44]. In LN mapping, however, there are inconsistencies [45-47] that may be attributable to inadequate standardization, training, and interpretation of micro-metastases and to skip metastases [43,48].

Survival in colon cancer is influenced by the presence of positive LNs and by the total number of positive LNs [32]. For Stage III tumors, the AJCC sub-classifies nodal staging into pN_1 _and pN_2_, based on the presence of ≥4 positive LNs [21]. In our analysis, the probability of having pN_2 _patients increased from 18% to 43% as the number of LNs examined increased from < 7 to ≥ 20. Similarly, there was increased disease-free survival as more LNs were examined from pN_1 _and pN_2 _patients [32]. The probability of missing a positive LN was 29.7%, 20.0%, and 13.6% when five, eight, and twelve LNs, respectively, were examined [49]. For node-positive patients, increased numbers of LN examination correlated with a lower LN ratio, which was associated with a better prognosis [37,50]. Although most of these investigations involved large sample sizes, the cutoff values differed; thus, further investigations were warranted.

## Conclusions

In summary, the mandatory 12 LNs examination recommended by different agencies (AJCC, ASCO, NCCN, etc.) did not demonstrate a significantly low risk of recurrence or survival benefit. Moreover, collection of ≥7 or ≥12 LNs had similar outcomes. Hence, a minimum of 6 LNs should be examined for adequate staging of Stage II and III colon cancer patients. Collection of ≥ 20 LNs, however, was associated with reduced risk of recurrence and improved survival for Stage II but not for Stage III colon cancer patients. Also, there is an improved survival with increased numbers of LNs harvested from Stage II and Stage III patients regardless of the cutoff points used. For Stage III tumors, every six additional LNs harvested resulted in identification of a positive LN. The probability of finding a pN_2 _patient increased with increasing numbers of LNs collected. Thus, to minimize stage misclassification and to aid in therapeutic decisions for colon cancer patients, the surgeons should perform more extensive lymphadenectomies and the pathologists should screen the surgical specimens diligently and examine as many LNs as possible. Furthermore, the findings from institutional studies, like ours, relate to the population of the serving area they represent. Thus, there may be geographic differences which can be addressed in future studies and minimized when one follows uniform treatment and pathology protocols.

## Financial and non-financial competing interests

The authors declare that they have no competing interests.

## Authors' contributions

CKS involved in conception, design, data collection, data assembly, data analysis, data interpretation, and manuscript writing. RBH involved in conception, design, data collection, data assembly, data analysis, data interpretation, and manuscript writing. NCJ involved in conception, design, data collection, data assembly, data analysis, data interpretation, and manuscript writing. VRK involved in data collection, data assembly, data analysis, and data interpretation. BZ involved in data analysis and data interpretation. JAP involved in provision of study patients, data collection, data assembly, data analysis, data interpretation, and manuscript writing. HLB involved in data collection, data assembly, data analysis, data interpretation, and manuscript writing. WEG involved in data analysis, data interpretation, and manuscript writing. IEE involved in data analysis and data interpretation. GPS involved in data analysis, data interpretation and manuscript writing. UM involved in administrative support, conception, design, provision of study patients, data collection, data assembly, data analysis, data interpretation, and manuscript writing. All authors read and approved the final manuscript.
